# A comparative study of three liquid platelet concentrates on human primary osteoblast activity: an *in vitro* study

**DOI:** 10.1590/1678-7757-2024-0575

**Published:** 2025-05-30

**Authors:** Vichuda CHATTRATHIKUL, Putida PINTHONGLOR, Chayarop SUPANCHART, Supatra SANGIN

**Affiliations:** 1 Chiang Mai University Faculty of Dentistry Department of Restorative Dentistry and Periodontology Thailand Chiang Mai University, Faculty of Dentistry, Department of Restorative Dentistry and Periodontology, Thailand.; 2 Chiang Mai University Faculty of Dentistry Department of Oral and Maxillofacial Surgery Thailand Chiang Mai University, Faculty of Dentistry, Department of Oral and Maxillofacial Surgery, Thailand.; 3 Chiang Mai University Center of Excellence in Materials Science and Technology Thailand Chiang Mai University, Center of Excellence in Materials Science and Technology, Thailand.

**Keywords:** Platelet-rich fibrin, Platelet-rich plasma, Liquid platelet concentrates, Osteoblast, Wound healing

## Abstract

**Objective:**

To investigate the effects of concentrated platelet-rich fibrin (C-PRF), injectable platelet-rich fibrin (i-PRF), and platelet-rich plasma (PRP) on cellular activity of human primary osteoblasts.

**Methodology:**

C-PRF, i-PRF, and PRP were prepared from five donors and pre-cultured in 5 mL of culture medium for three days. Human primary osteoblasts were seeded and cultured with 20% conditioned medium derived from the three platelet concentrates. Then, osteoblast viability was assessed at 24 h; proliferation at one, three, and five days; differentiation at seven days; mineralization at 14 days; and gene expression *RUNX* family transcription factor 2 (*RUNX2*), alkaline phosphatase, biomineralization associated (*ALPL*), collagen type I alpha 1 chain (*COL1A1*), and osteocalcin (*OCN*) at three and 14 days were investigated.

**Results:**

Osteoblasts cultured with C-PRF, i-PRF, and PRP demonstrated excellent biocompatibility. Proliferation was significantly higher in all platelet concentrates compared to the controls at one, three, and five days, with no significant differences among them, except on day one. Alkaline phosphatase and Alizarin Red S staining were significantly higher in the C-PRF and i-PRF groups compared to the PRP and control groups. However, *RUNX2*, *ALPL*, *COL1A1*, and *OCN* mRNA levels did not differ significantly among the three platelet concentrates throughout the study period.

**Conclusion:**

Our study indicates that the three liquid platelet concentrates enhance human osteoblast activity. C-PRF and i-PRF promoted greater differentiation and mineralization than PRP. These findings show that all liquid platelet concentrates positively influence human osteoblast proliferation and differentiation, making them suitable for clinical applications requiring bone regeneration.

## Introduction

Platelet concentrates have been widely used in regenerative medicine and dentistry for over three decades due to their autologous growth factors, which enhance natural healing and tissue regeneration.^1^ Platelet-rich plasma (PRP), the first-generation platelet concentrate, was introduced by Marx, et al.^2^ (1998) for bone reconstruction. Its preparation is complex, often requiring anticoagulants and coagulation activators. While PRP benefits osteoblast proliferation and differentiation, its clinical efficacy remains inconsistent due to anticoagulant-induced cytotoxicity and impaired clotting or healing.^3,4^

Therefore, Choukroun, et al.^5^ (2001) developed leukocyte platelet-rich fibrin (L-PRF), a second-generation platelet concentrate obtained after a single-step centrifugation process (700 g for 12 min) without anticoagulants. This technique forms a fibrin clot that traps platelets, leukocytes, cytokines, and stem cells, ensuring a sustained release of key growth factors, including transforming growth factor-β (TGF-β), bone morphogenetic protein (BMP), platelet-derived growth factors (PDGFs), vascular endothelial growth factor (VEGF), and epidermal growth factor (EGF). These factors are crucial in bone repair, as they promote angiogenesis, cell proliferation, differentiation, extracellular matrix synthesis, and inflammatory cell chemotaxis.^6–8^ L-PRF has demonstrated superior wound healing and regenerative benefits over PRP in medical and dental research.^9^ The global adoption of this open-access method, combined with advancements in protocols and centrifuge techniques, has led to the advance of alternative autologous PRF derivatives.

Choukroun and Ghanaati^10^ (2018) developed injectable PRF (i-PRF) by reducing the relative centrifugal force (RCF) and time (60 g for three min) using plastic tubes. This approach enhanced platelet, leukocyte, and growth factor concentrations. Wend, et al.^11^ (2017) demonstrated that i-PRF prepared with low-speed centrifugation significantly increased PDGF-BB, TGF-β1, VEGF, and EGF levels compared to high-speed centrifugation. Applications of i-PRF have expanded across various dental fields, including periodontal and bone regeneration, orthodontic tooth movement, temporomandibular joint disorders, and endodontics, either alone or combined with biomaterials.^12^

Although previous *in vitro* studies have reported that i-PRF enhances osteoblast proliferation, differentiation, and mineralization on tissue culture more effectively than PRP, A-PRF, L-PRP, and freeze-dried homologous PRP,^13,14^ Miron, et al.^15^ (2019) reported that the original i-PRF protocols failed to fully concentrate platelets in the upper plasma layer, leaving most platelets and leukocytes in the lower layers. For this reason, Miron, et al.^16^ (2020) introduced concentrated PRF (C-PRF), a liquid PRF obtained using a novel harvesting technique from the 0.5 mL buffy coat layer. This method follows the standard L-PRF protocol, but uses a plain plastic tube for centrifugation, preventing activation of the coagulation cascade and enabling the plasma to remain liquid for approximately 15–20 min, unlike the glass or silica-coated tubes used in L-PRF. As a result, this collection technique led to a 10-fold increase in platelet and white blood cell concentrations within the 0.3–0.5 mL buffy coat layer, located directly above the red blood cell corpuscle, significantly surpassing the 2–4-fold increase observed in i-PRF. Fujioka-Kobayashi, et al.^17^ (2020) reported a 2–3-fold increase in PDGF-AA, TGF-β, and EGF release in C-PRF over 10 days, better enhancing fibroblast migration, proliferation, and collagen I synthesis compared to i-PRF.

Research on the osteogenic potential of liquid PRF remains limited. A previous *in vitro* study found that combining i-PRF with bone substitutes improved osteoblast metabolism, increasing alkaline phosphatase (ALP), BMP-2, and osteocalcin (OCN) expression.^18^ Additionally, i-PRF was shown to promote greater osteoblast differentiation and ALP activity than L-PRF.^19^ However, no study has directly compared PRP, i-PRF, and C-PRF. This study aims to evaluate their effects on human primary osteoblast activity *in vitro*.

## Methodology

All procedures involving human participants in this study were approved by the Human Experimentation Committee at the Office of Research Ethics, Faculty of Dentistry, Chiang Mai University (Approval No. 49/2022).

### Preparation of platelet concentrates

Blood samples were collected from five volunteers (21–40 years old, systemically healthy, non-smokers, and medication-free), who provided informed consent. Each volunteer donated a total of nine blood collection tubes from venous blood using a 24-gauge butterfly needle, with three tubes allocated to each of the three test groups: PRP, i-PRF, and C-PRF. The fixed-angle centrifuge, the IntraSpin^®^ Device (IntraLock, Boca Raton, FL, USA), was used for all protocols. PRP was prepared following a previously described protocol.^13^ Briefly, 10 mL of blood was collected in K2-ethylenediaminetetraacetic acid (K2-EDTA) tubes (Becton, Dickinson and Company, Franklin Lakes, NJ, USA) and centrifuged at 900 g (3,100 rotations per min [RPM]) for five min to remove red blood cells (RBCs). Next, a second centrifugation at 2000 g (4,700 RPM) for 15 min was performed using plastic polyethylene terephthalate (PET) tubes (Bio-PRF, Jupiter, FL, USA) without chemical additives to separate 1 mL of PRP from the platelet-poor plasma at room temperature. To prepare i-PRF and C-PRF following Miron, et al.^16^ (2020), 10 mL of blood was collected in plastic PET tubes and centrifuged at an RCF-max of 60 g (800 RPM) for three min for i-PRF or an RCF-max of 700 g (2,700 RPM) for 12 min for C-PRF, both at room temperature. The upper 1 mL layer, including the buffy coat located directly above the RBC layer, was collected. Finally, 1 mL of each platelet concentrate, under sterile conditions (airflow cabinet), was transferred to six-well culture dishes containing 5 mL of Dulbecco's modified Eagle medium (DMEM; Gibco BRL, Grand Island, NY, USA) with 1% penicillin/streptomycin (Gibco RBL) and incubated for three days at 37°C in a humidified 5% CO_2_ atmosphere for further processing.^13,17^

### Isolation of human osteoblasts

Human osteoblasts were obtained from the jawbone during orthognathic surgery on five healthy patients (18–35 years old), who provided written informed consent. Cell isolation was conducted according to the previously described protocol.^20^ Briefly, the harvested bone specimens were washed several times with 4-(2-hydroxyethyl)-1-piperazineëthanesulfonic acid-buffered saline to remove blood cells until the buffer became clear, then cut into smaller pieces. A sequential digestion process was performed to promote osteoblast migration by incubating the bone fragments twice in a 1 mg/mL Collagenase/Dispase® solution (Sigma-Aldrich, St. Louis, MO, USA) in DMEM, followed by a single incubation in EDTA-trypsin (Gibco BRL), and a final incubation in Collagenase/Dispase^®^ solution. Each incubation step was carried out at 37°C for 30 min. The supernatant from each incubation was collected into a 50-mL centrifuge tube containing an equal volume of growth medium (DMEM with 10% fetal bovine serum [FBS; Gibco BRL] and 1% penicillin/streptomycin). Then, the mixture was centrifuged at 650 g for five min (Centrifuge 5702 RH, Eppendorf, Hamburg, Germany), and the supernatant was carefully removed. The cell pellet was then resuspended in enriched DMEM supplemented with 20% FBS (Gibco RBL) and 1% penicillin/streptomycin, then placed in a 75-cm^2^ culture flask. The cells were incubated at 37°C in a humidified 5% CO_2_ atmosphere, with the medium replaced every three to four days. Upon reaching 80%–90% confluence, they were trypsinized and passed into growth media. Cells from passages three to five were used for experiments.

### Cell culture

The three platelet concentrates were incubated for three days, and 20% of the conditioned media was collected for future experiments.^13,17^ Human osteoblasts were seeded in growth media supplemented with 20% conditioned media for viability and proliferation studies. Additionally, cells were seeded in osteogenic differentiation media (ODM; DMEM, 10% FBS, 1% penicillin/streptomycin, 10 mM β-glycerophosphate, 50 μg/mL ascorbic acid, and 10 nM dexamethasone [Sigma–Aldrich])^21^ with 20% conditioned media for alkaline phosphatase (ALP) and Alizarin Red S (ARS) staining, as well as real-time reverse transcription-quantitative polymerase chain reaction (RT-qPCR) analysis. Experiments were divided into four groups: control, C-PRF, i-PRF, and PRP. In the control group, cells were cultured in growth media or ODM without 20% conditioned media. Each group was assigned three wells, and for experiments exceeding five days, the media were changed twice per week.

### Cell viability

Human osteoblasts were evaluated 24 h after cell seeding in 96-well culture plates at a density of 7,500 cells per well using the 3-(4,5-dimethylthiazol-2-yl)-2,5-diphenyltetrazolium bromide (MTT) assay (Sigma-Aldrich). MTT (20 μL, 5 mg/mL) was added to each well and incubated for 4 h. After discarding the media, 200 μL of dimethyl sulfoxide (Sigma–Aldrich) was added per well to dissolve the formazan, followed by gentle shaking at room temperature for 10 min. Then, the plates were read at 540 nm with a reference wavelength of 690 nm using a microplate reader (Sunrise™; Tecan, Männedorf, Switzerland).

### Cell proliferation

Human osteoblasts were seeded in 96-well plates at a density of 7,500 cells per well and quantified using a bromodeoxyuridine (BrdU) colorimetric immunoassay (Roche Diagnostics, Indianapolis, IN, USA) at one, three, and five days. At each designated time point, BrdU labeling solution was added and incubated for 15 h. After media removal, cells were fixed with 200 μL of FixDenat solution for 30 min at room temperature to denature DNA. A 100-μL aliquot of anti-BrdU was added and incubated at room temperature for 90 min. After three washes with 300 μL of washing buffer to remove excess antibody, 100 μL of substrate solution was added and incubated in the dark for 30 min at room temperature. The reaction was stopped with 25 μL of sulfuric acid, and the cells were measured using a microplate reader at 450 nm with a reference wavelength of 690 nm.

### Differentiation and mineralization assays

#### ALP staining

After seven days, human osteoblasts (7,500 cells/well) seeded in 24-well plates were fixed with 4% paraformaldehyde in phosphate-buffered saline (PBS) at 10°C for 15 min, then rinsed twice with 0.5 mL of PBS. After removing the buffer, the cells were stained with 0.5 mL of a mixture containing CHAP buffer (100 mM Tris, pH 9.5, 100 mM NaCl, and 50 mM MgCl_2_ in distilled water) and nitroblue tetrazolium chloride/5-bromo-4-chloro-3-indolyl phosphate toluidine salt (NBT/BCIP; Roche Diagnostics), incubated at in the dark at room temperature for 60 min, and washed twice with PBS for five min. ALP staining was imaged using a stereomicroscope (E-330; Olympus, Inc., Tokyo, Japan), and the stained area percentage was quantified using ImageJ software (LOCI, University of Wisconsin, USA).

#### ARS staining

After 14 days, human osteoblasts (7,500 cells/well) seeded in 24-well plates were fixed with 4% paraformaldehyde in PBS at 10°C for 15 min, then rinsed twice with 0.5 mL of PBS. The cells were then stained with a 2% ARS solution (Sigma-Aldrich), pH 4.1–4.3, for 45 min at room temperature. Excess dye was removed with multiple distilled water rinses, followed by a final rinse with 0.5 mL of PBS to stop the reaction. ARS staining was imaged by a stereomicroscope, and the stained area percentage was quantified with ImageJ software.

To quantify staining, 0.2 mL of 10% acetic acid was added to the ARS-stained cells per well and incubated for 30 min at room temperature with shaking to solubilize red stains. The cells were scraped and the mixture was transferred to 1.5-mL centrifuge tubes. After vortexing for 30 sec, the tubes were heated at 85°C for 10 min, cooled on ice for five min, and centrifuged at 12,000 g for 15 min (Centrifuge 5415 R, Eppendorf). Subsequently, 100 μL of the supernatant was transferred to a 96-well black plate with a clear bottom, neutralized with 30 μL of 10% ammonium hydroxide, and analyzed colorimetrically at 405 nm, with a reference wavelength of 690 nm.^22^

### RNA extraction and RT-qPCR

**Figure 1:**
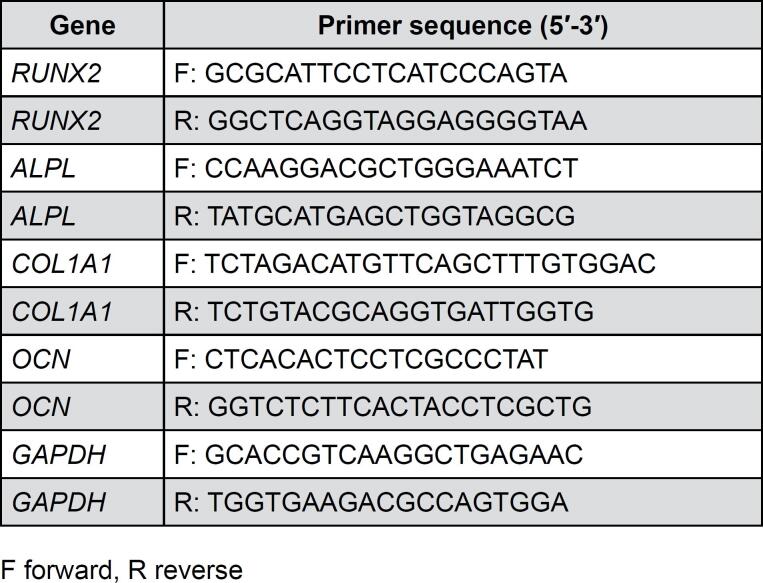
Primer sequences used for real-time PCR

RT-qPCR analysis was performed using cells plated at a density of 10,000 per well in 6-well plates. The expression levels of RUNX family transcription factor 2 *(RUNX2)*, alkaline phosphatase, biomineralization associated *(ALPL)*, collagen type I alpha 1 chain *(COL1A1)*, and osteocalcin *(OCN)* were quantified. At three and 14-days post-stimulation, total RNA was extracted from human osteoblasts using the Illustra RNAspin Mini Isolation Kit (GE Healthcare, Little Chalfont, Buckinghamshire, UK) following the manufacturer's protocol. RNA concentration was determined using a NanoDrop™ 2000/2000c spectrophotometer (Thermo Fisher Scientific, Rochester, NY, USA) at 260 nm and 280 nm. Then, the RNA was reverse-transcribed into complementary DNA using the iScript™ Reverse Transcription Supermix (Bio-Rad, Hercules, CA, USA). Primer sequences *(RUNX2*, *ALPL*, *COL1A1*, *OCN*, and glyceraldehyde-3-phosphate dehydrogenase [*GAPDH* as a housekeeping gene]) are listed in Figure 1.^13^ RT-qPCR was performed using the iTaq™ Universal SYBR Green Supermix (Bio-Rad) with a LightCycler 480 instrument II (Roche, Rotkreuz, Switzerland).

### Statistical analysis

All experiments were performed in triplicate. Means and standard errors (SEs) were calculated and statistically compared using one-way ANOVA. Tukey's test was applied for equal variance, and Dunnett's T3 test was used for unequal variance using SPSS (version 17; SPSS Inc., Chicago, IL, USA). Statistical significance was set at *p<0.05*.

## Results

The blood donors’ mean age was 29.40±3.65 years, ranging from 25 to 35 years, while osteogenic donors had a mean age of 23.80±3.70 years, ranging from 21 to 30 years. Each group had an equal gender distribution: two males (40%) and three females (60%). Sociodemographic details are shown in Table 1.

**Table 1:** Sociodemographic characteristic of blood and osteogenic donors

Blood donors	Gender	Age	Osteogenic donor	Gender	Age
Donor 1	male	30	Donor 1	male	21
Donor 2	female	35	Donor 2	female	24
Donor 3	male	25	Donor 3	female	30
Donor 4	female	29	Donor 4	female	23
Donor 5	female	28	Donor 5	male	21
**Age (Y)**	Mean±SD	29.40±3.65	**Age (Y)**	Mean±SD	23.80±3.70
	Range	25-35		Range	21-30
**Gender (n)**	male	2 (40%)	**Gender (n)**	male	2 (40%)
	female	3 (60%)		female	3 (60%)

### Osteoblast viability

**Figure 2:**
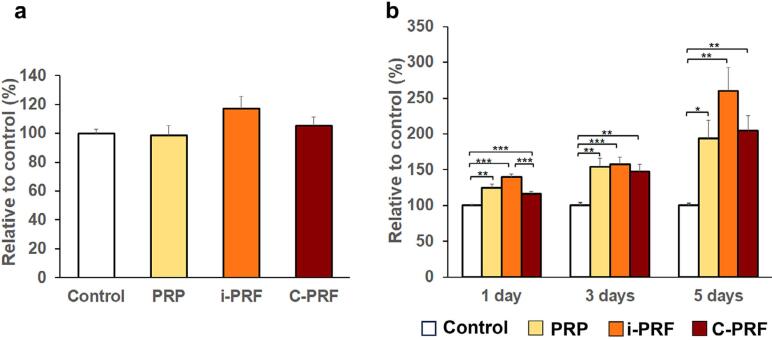
Mean percentages of (a) cell viability after 24 h and (b) cell proliferation at one, three, and five days compared to the control group (set to 100). Error bars = SE; *n* = five per group; *p<0.05; **p<0.01; ***p<0.001

The effects of PRP, i-PRF, and C-PRF on the viability of human osteoblasts were examined (Figure 2a). Over 95% of cells in each group survived, with no significant differences among them or compared to the control. Therefore, PRP, i-PRF, and C-PRF demonstrated excellent biocompatibility and exhibited minimal to no cytotoxicity with this *in vitro* model.

### Osteoblast proliferation

The BrdU assay revealed that PRP, i-PRF, and C-PRF significantly enhanced cell proliferation compared to the control at all time points (one, three, and five days). While no significant differences were observed among platelet concentrates, i-PRF promoted higher cell proliferation than C-PRF on day one (Figure 2b).

### Differentiation and mineralization assays:

#### ALP staining

**Figure 3:**
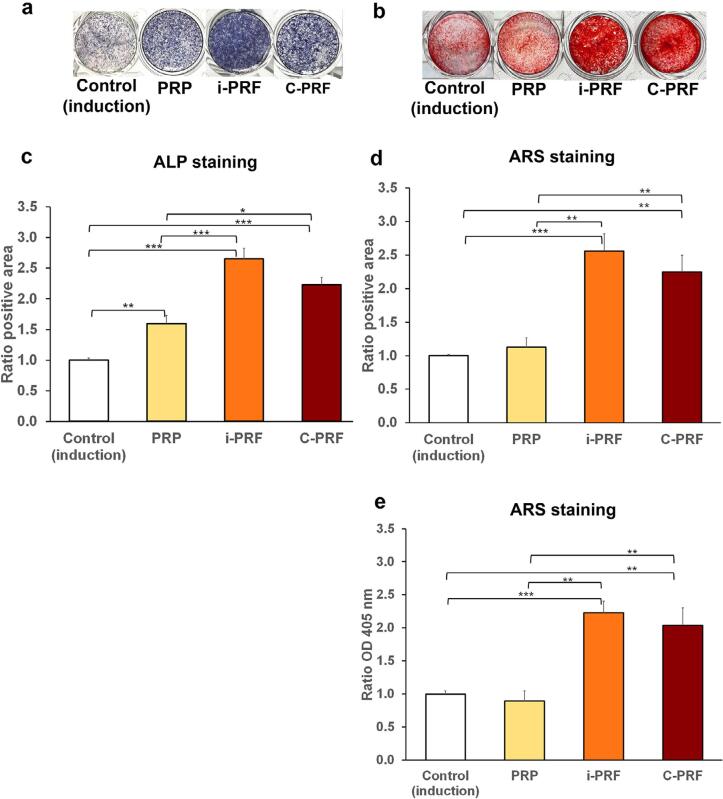
Effects of PRP, i-PRF, and C-PRF on the differentiation and mineralization of primary human osteoblasts. Representative images of (a) alkaline phosphatase (ALP) staining at seven days and (b) Alizarine Red S (ARS) staining at 14 days. The mean ratios of the positive area for (c) ALP staining in (a) and (d) ARS staining in (b) were quantified by ImageJ analysis. (d) The mean OD ratios at 405 nm after solubilization of ARS staining deposited in (e). The control was set to 1. Error bars = SE; *n* = five per group; *p<0.05; **p<0.01; ***p<0.001

At seven days, ALP staining was significantly stronger in osteoblast cultures treated with i-PRF or C-PRF compared to PRP or the control (Figure 3a). Consistently, the mean ratio of the positive area was notably higher with all three platelet concentrates than with the control. Among the three platelet concentrates, ALP staining was stronger in cell cultures treated with i-PRF or C-PRF than with PRP (Figure 3c).

#### ARS staining

At 14 days, ARS staining intensities and mean positive area ratios were significantly greater for osteoblasts cultured with i-PRF or C-PRF than with PRP or the control (Figure 3b and d). Similarly, the mean optical density (OD) ratios at 405 nm were significantly higher for cells cultured with i-PRF or C-PRF than with PRP or the control (Figure 3e). These findings suggest that osteoblasts treated with i-PRF or C-PRF better promoted the formation of mineralized nodules compared to PRP or the control.

### Gene expression

**Figure 4:**
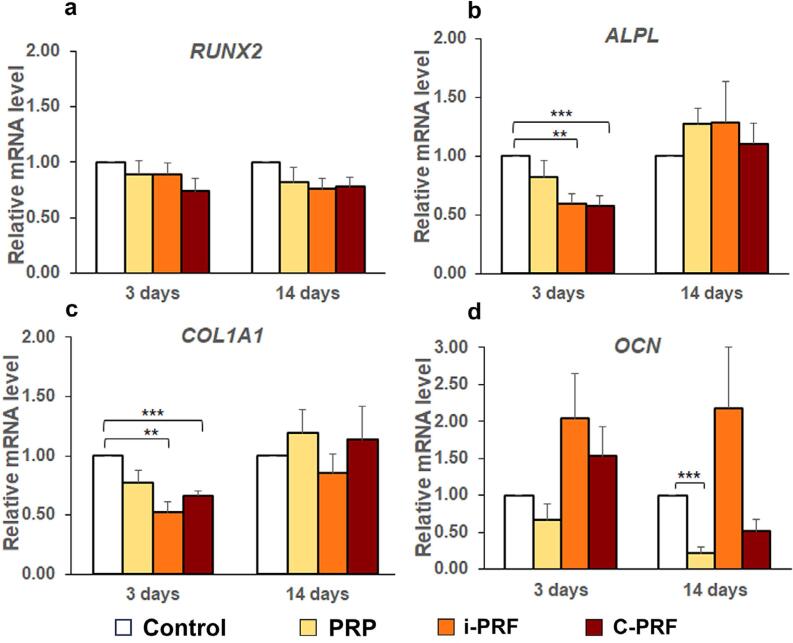
Mean mRNA levels of (a) *RUNX2*, (b) *ALPL*, (c) *COL1A1*, and (d) *OCN* relative to *GAPDH* as the control in primary human osteoblasts treated with PRP, i-PRF, and C-PRF compared to the control group (set to 1) at three and 14 days. Error bars = SE; *n* = five per group; **p<0.01; ***p<0.001

The mRNA levels of osteoblastic differentiation markers (*RUNX2*, *ALPL*, *COL1A1*, and *OCN*) did not differ significantly among PRP, i-PRF, and C-PRF at three and 14 days (Figure 4a-d). However, mRNA levels of *ALPL* and *COL1A1* were significantly higher in the control group than in the i-PRF and C-PRF groups at three days (Figure 4b and c). Additionally, the mRNA levels of *OCN* were significantly higher in the control group than in the PRP group at 14 days (Figure 4d).

## Discussion

This *in vitro* study evaluated three different injectable PRF preparation protocols (C-PRF, i-PRF, PRP) regarding their biological effect on human primary osteoblast. Cell viability, proliferation, differentiation, mineralization, and gene expression of osteoblastic differentiation markers (*RUNX2*, *ALPL*, *COL1A1*, and *OCN*) were analyzed.

In the first experiment examining osteoblast viability, PRP, i-PRF, and C-PRF demonstrated excellent biocompatibility (>98%), meeting the standards of minimal to non-toxic effects per ISO 10993-5: 2009(E) standard. Similarly, Wang, et al.^13^ (2018) found that both i-PRF and PRP sustained osteoblast viability above 95%. Moreover, Fujioka-Kobayashi, et al.^17^ (2020) observed that gingival fibroblasts cultured with i-PRF and C-PRF exhibited high percentages of living cells (>95%).

Furthermore, all platelet concentrates significantly increased cell proliferation compared to the control. The three platelet concentrates did not differ significantly, except on day one, when proliferation was higher with i-PRF than with C-PRF. Consistently, Wang, et al.^13^ (2018) reported that both i-PRF and PRP significantly increased the proliferation of human primary osteoblasts compared to the control at three and five days, with proliferation higher with i-PRF among all groups at five days. According to Ogino, et al.^23^ (2006), the number of platelets and the levels of growth factors in platelet concentrates contributed to osteoblast proliferation. Miron, et al.^24^ (2017) discovered that i-PRF released higher levels of PDGF-AA, PDGF-AB, and insulin like growth factor 1 (IGF-1) than PRP after three days. This finding suggests these growth factors are crucial in osteoblast proliferation within i-PRF. Among them, PDGF exhibited the most significant effect on cell proliferation, followed by TGF-β and IGF-1.^23^ In contrast, Fujioka-Kobayashi, et al*.*^17^ (2020) found that C-PRF released higher levels of PDGF-AA, PDGF-AB, PDGF-BB, and TGF-β than i-PRF after three days. However, it is important to note that, in their study, both C-PRF and i-PRF were prepared using a horizontal centrifuge, while in our study, they were prepared using a fixed-angle centrifuge. The type of centrifuge may impact the accumulation of cells and levels of growth factors.^15^

When evaluating osteoblastic differentiation, cells treated with i-PRF or C-PRF had significantly greater mean ratios of positive area compared to those treated with PRP or the control. These findings are consistent with Wang, et al.^13^ (2018), who found that osteoblasts treated with i-PRF had significantly higher ALP activity than those treated with PRP or the control at seven days. Miron, et al.^24^ (2017) reported that i-PRF released significantly higher levels of IGF-1 than PRP. Similarly, Fujioka-Kobayashi, et al*.*^17^ (2020) found that IGF-1 release was higher from i-PRF than from C-PRF, despite using a horizontal centrifuge. Therefore, the higher levels of IGF-1 in i-PRF and C-PRF may enhance osteoblastic differentiation.^25^

Mineralization showed the same trend, with osteoblasts cultured in i-PRF and C-PRF exhibiting notably higher mean positive area and OD ratios for ARS staining than PRP or the control at 14 days. Similarly, Wang, et al.^13^ (2018), reported significantly greater ARS staining in osteoblasts with i-PRF than with PRP. In our study, i-PRF and C-PRF had greater positive effects on osteoblast differentiation and mineralization than PRP.

In the initial stages of hard tissue formation, ALP within matrix vesicles hydrolyzes inorganic pyrophosphate to produce inorganic phosphate (Pi). This Pi combines with calcium ions (Ca^2+^) to facilitate the continuous formation of hydroxyapatite crystals, aiding mineralized tissue development.^26^ The presence of EDTA in PRP may compete with Pi in the extracellular matrix to chelate Ca^2+^. This competition potentially lowered ARS staining in the PRP group compared to the C-PRF and i-PRF groups.^27^

The mRNA levels of *RUNX2, ALPL, COL1A1,* and *OCN* did not differ significantly among the three platelet concentrates at three and 14 days. This is consistent with Kosmidis, et al.^19^ (2023), who reported no significant differences in the mRNA levels of these genes among PRF-stimulated cultures at the same time points. However, they noted that i-PRF significantly increased ALP activity compared to the control, promoting greater osteoblast-like cell differentiation. Similarly, this study found higher *RUNX2* expression in the control group compared to the platelet concentrate groups, while ALP staining was more pronounced in the i-PRF group than in the control. As an early marker of osteoblast differentiation, *RUNX2* expression is upregulated in pre-osteoblasts, peaks in immature osteoblasts, and is downregulated in mature osteoblasts.^28^ This suggests that osteoblast differentiation from pre-osteoblasts to mature osteoblasts was more advanced in the three platelet concentrate groups than in the control group. However, *ALPL* mRNA levels at three and 14 days did not correlate with ALP staining results at seven days with the three platelet concentrates, possibly due to timing differences in result interpretation. *COL1A1* serves as a late osteogenic marker that is crucial for mineralization. Its expression peaks after three weeks.^29^ *OCN* mRNA levels are associated with late osteoblast differentiation and mineralization.^30^ At 14 days, *OCN* expression did not correlate with ARS staining, and levels were lower in the C-PRF and PRP groups compared to the control. These results contrast with Wang, et al.^13^ (2018), who reported significantly higher mRNA levels of *RUNX2, ALPL, COL1A1,* and *OCN* in i-PRF and PRP compared to control at 14 days.

The three platelet concentrates promoted osteoblast proliferation, particularly i-PRF on day one. Their impact on differentiation phenotypes (ALP activity and Alizarin staining) may be due to growth factors released from the platelet concentrates following proliferation.^31^ Nevertheless, gene expression may not fully explain differentiation phenotypes, as it precedes phenotypic changes.

A key strength of this *in vitro* study is its direct comparison of C-PRF, i-PRF, and PRP on human primary osteoblast activity. Previous research indicates that C-PRF accumulates higher concentrations of cells and growth factors, providing greater support for fibroblast function compared to i-PRF.^16,17^ Therefore, it was hypothesized that C-PRF would provide better support for osteoblasts. However, our study found no significant differences in cell proliferation, gene expression, osteoblastic differentiation, or mineralization among the three platelet concentrates. These findings may be influenced by factors such as assessment timing, study design, cell culture conditions, and individual variability.

One limitation of this study is that we did not use horizontal centrifugation to prepare C-PRF and i-PRF, as done in a previous study.^17^ We prepared three liquid platelet concentrates using a fixed-angle centrifuge (IntraSpin^®^ Device) and C-PRF protocol was followed the original protocol by Miron, et al.^16^ (2020) and Quirynen, et al.^32^ (2023), using a fixed-angle centrifuge at 700 g RCF-max (~400 g RCF-clot) for 12 min. Notably, the C-PRF preparation protocol varies across studies. While Miron, et al.^16^ (2020) used a fixed-angle centrifuge under the same conditions as our study, Fujioka-Kobayashi, et al.^17^ (2020) employed a horizontal centrifuge at 3000 g for eight minutes. Other studies have reported variations in centrifugation parameters, including centrifuge type, spinning time, speed (RPM), and g-force.^33–35^ Previous studies have shown that centrifuge equipment, tube selection, and centrifugation protocols can influence the accumulation of cells and growth factors.^15,36^

The development of liquid PRF was mainly aimed at enhancing regenerative potential, improving handling properties, and optimizing growth factor release compared to standard L-PRF. Its injectable form, superior biological activity, and versatility make it a preferred choice in regenerative applications.^24,37,38^ Further clinical studies are needed to elucidate the comparative advantages of these liquid platelet concentrates and their potential benefits in wound healing.

## Conclusions

The findings of this *in vitro* study indicate that the three liquid platelet concentrates enhance human osteoblast activity. However, C-PRF and i-PRF promoted differentiation and mineralization better than PRP.

## Data Availability

All data generated or analyzed during this study are included in this published article.
